# Effects of acute cardiovascular exercise and coordinative motor activity on verbal functions and attention

**DOI:** 10.1007/s10339-026-01380-2

**Published:** 2026-07-29

**Authors:** Mira Fischer, Veit S. Kraft, Joachim Hermsdörfer

**Affiliations:** 1 Department of Neurology, LMU University Hospital, Ludwig-Maximilians-Universität München, Munich, Germany; 2 German Center for Neurodegenerative Diseases, Munich, Germany; 3 Chair of Human Movement Science, TUM School of Medicine and Health, Technical University of Munich, Munich, Germany

**Keywords:** Cognition, Memory, Verbal functions, Acute exercise, Exercise modality, Language

## Abstract

Language is a cognitive domain essential for communication; however, its responsiveness to acute physical exercise or activity is unexplored. This quasi-randomized trial examined the effects of different intervention modalities on verbal functions and attention. Ninety-eight participants were assigned to high-intensity interval training (HIIT), coordinatively demanding motor activity (COOR), or a sedentary control group (CG). A pre–post design with a 24-hour follow-up assessed semantic (VFsem) and category-switching verbal fluency (VFsw), verbal learning, and attention. No group × time effects were observed for verbal learning encoding (*p* = .258); however, HIIT showed a steeper learning slope compared to CG (*p* = .044). Following an interference list, proactive interference was unaffected (*p* = .428), whereas retroactive interference differed significantly between groups (*p* = .019), with CG showing lower susceptibility than both intervention groups. No group differences were found for consolidation after 20 min or 24 h (*p*’s ≥ .271). Semantic verbal fluency improved significantly following HIIT relative to CG (*p* = .029), with no significant effects in VFsw (*p* = .221) or attention (*p* = .187). We conclude an intensity-dependent pattern following the interventions, manifesting as enhanced semantic lexical retrieval and an exploratory finding of a steeper encoding following HIIT, compared to CG, with COOR in between, accompanied by an increased susceptibility to retroactive interference.

**Supplementary Information:**

The online version contains supplementary material available at https://doi.org/10.1007/s10339-026-01380-2.

## Introduction

Language serves as the primary medium of human communication and learning, playing a vital role in social interactions, education, and professional environments (Kovarsky et al. 1993). Language comprehension and production processes are highly complex, requiring the seamless integration of phonological, semantic, and syntactic information. These are mediated by executive functions, including cognitive flexibility, attention, inhibition, and working memory, which enable efficient language use and information processing (Roelofs and Piai 2011).

Given the importance of cognitive abilities such as verbal fluency, verbal comprehension, memory, and perceptual speed in daily life (Lang et al. 2010), identifying and implementing effective strategies to enhance cognitive functions in general and verbal abilities in particular is of considerable scientific and practical interest.

Physical exercise and activity have gained increasing attention as a lifestyle-based tool for enhancing cognitive function across the lifespan (Hsieh et al. 2020). While chronic exercise interventions have been extensively studied, acute exercise has also been shown to transiently affect cognition, including reaction time, executive function, and memory (Ai et al. 2021; Y. K. Chang et al. 2012; Roig et al. 2016). The magnitude of this effect, however, varies and may depend on the intensity, timing, duration, and modality of the intervention, as well as on task characteristics and participant-related factors (Garrett et al. 2024; Roig et al. 2016). In particular, high-intensity cardiovascular exercise has been associated with exercise-induced neurochemical and vascular changes that may support memory, attention, and lexical retrieval (Basso and Suzuki 2017; Roig et al. 2016).

Despite growing interest in exercise-induced cognitive enhancement, the acute effects of different activity modalities on verbal functions remain insufficiently understood. Existing evidence suggests that cardiovascular exercise may facilitate aspects of verbal learning and language acquisition. For example, a single sprint training session (Winter et al. 2007), learning during cycling (Liu et al. 2017) and high-intensity cardiovascular exercise performed in close temporal proximity to vocabulary learning (Frith et al. 2017; Salis 2013) have been linked to improved verbal learning or retention. These findings suggest that acute cardiovascular exercise may be particularly relevant when its transient physiological effects overlap with encoding or early consolidation processes. Thereby, encoding describes the initial acquisition of new information during learning, whereas consolidation refers to the stabilization and transfer of information into long-term memory for later retrieval (Squire et al. 2015).

In contrast to cardiovascular exercise, coordinatively demanding motor activity has received comparatively little attention in relation to verbal functions. Such activity may be relevant because cognitive-motor demands, including balance, bilateral coordination, hand-eye coordination, and cognitive-motor interference, engage neural processes that partly overlap with those involved in executive control, attention, and memory (Ludyga et al. 2020; Pesce et al. 2021). This assumption is consistent with the view that cognitively demanding motor activity may support cognition through task complexity and prefrontal engagement rather than primarily through metabolic load (Pesce et al. 2021). Similarly, the Adaptive Capacity Model proposes that cognitive benefits may be especially likely when physical activity is combined with cognitive demands, as would have occurred during evolutionarily relevant activities such as foraging, navigation, and decision-making during locomotion (Raichlen and Alexander 2017; Raichlen and Polk 2013). Accordingly, coordinative motor activity may provide a theoretically meaningful contrast to high-intensity cardiovascular exercise because it emphasizes movement complexity and cognitive-motor integration rather than cardiovascular intensity.

However, it is important to note that the cardiovascular and coordinative conditions differ in more than one respect. Whereas the cardiovascular condition is characterized by a high physiological load, the coordinative condition places greater demands on movement complexity and cognitive-motor processing. Consequently, potential differences between these conditions cannot be attributed solely to either intensity or coordinative demands. Nevertheless, comparing these modalities provides an opportunity to explore whether acute changes in verbal and attentional performance are more strongly associated with cardiovascular exertion or with cognitively demanding motor activity. This distinction is particularly relevant given that it remains unclear to what extent potential cognitive benefits of coordinative demands occur independently of, or in combination with, higher levels of physical exertion.

Regarding participants’ characteristics, most studies focus on children or older adults, where low to moderate effect sizes were observed (Erickson et al. 2019; Ludyga et al. 2020). Young adults remain comparatively underrepresented, although verbal learning, verbal fluency, and attention are highly relevant for academic and professional performance. Understanding how different acute activity modalities affect these functions in young adults may therefore provide practically relevant insights for brief, everyday interventions.

The present study aimed to examine the acute effects of cardiovascular exercise and coordinatively demanding motor activity, compared with a verbally inert sedentary control task, on verbal fluency, verbal learning, and sustained attention. The coordinative condition comprised balance, bilateral coordination, hand–eye coordination, and cognitive-motor interference tasks while imposing only a low cardiovascular load. Based on the neurochemical and vascular mechanisms associated with high-intensity cardiovascular exercise, as well as the proposed engagement of cognitive control processes during coordinatively demanding motor activity, we hypothesized that cardiovascular exercise would lead to the greatest improvements in verbal functions and attention compared with the sedentary control task, with coordinatively demanding motor activity lying in between. By directly contrasting a high-intensity cardiovascular condition with a low-intensity but coordinatively complex motor condition, this study addresses whether acute changes in verbal and attentional performance are more consistent with cardiovascular-intensity or cognitive-motor complexity conditions.

## Methods

### Study design

**Fig. 1:**
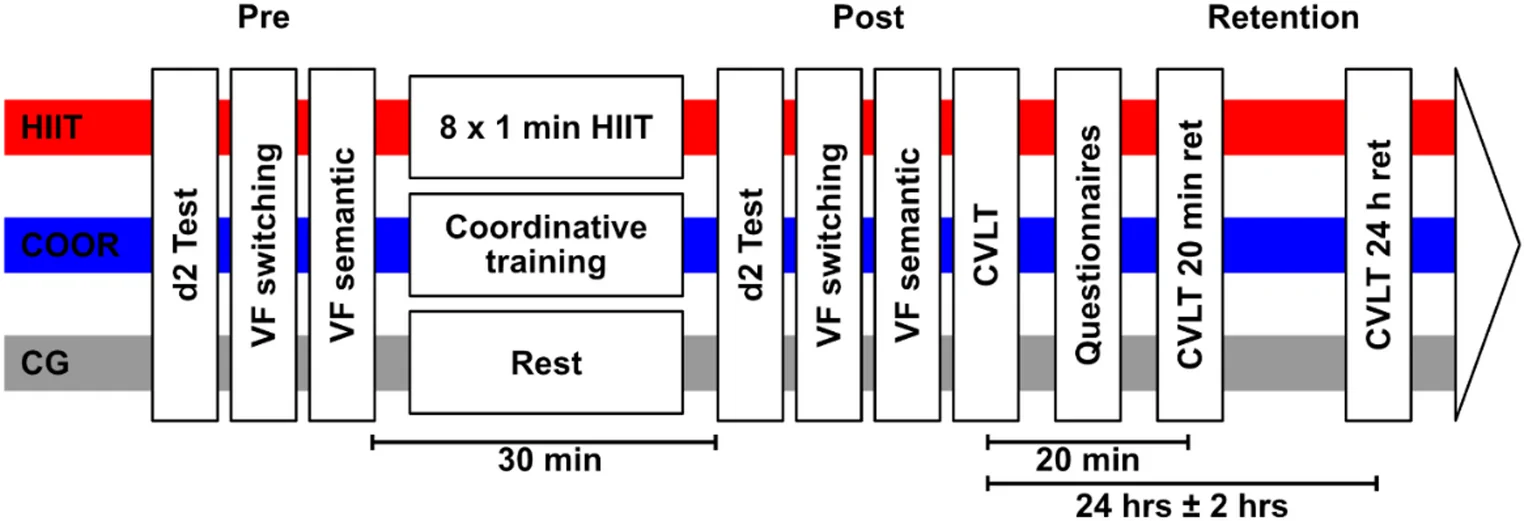
Quasi-randomized controlled trial design with three groups (HIIT: high-intensity interval training, COOR: coordinative training, and CG: control group). A pre-post test design was assessed for d2, and verbal fluency semantic (VFsem) and category switching (VFsw). Verbal learning using an adapted version of the California Verbal Learning Test (CVLT) was administered following the intervention with a 20 min and 24 h recall test. The 20 min retention break was used to assess questionnaires

The study employed a between-subject design with quasi-randomized group allocation of three groups: a control group (CG), a coordinative training group (COOR), and a high-intensity interval training (HIIT) group. Participants were assigned to one of these three groups in a rotating sequence based on their order of random arrival to the laboratory. When two participants were tested simultaneously, both were allocated to the same group for logistical reasons. Consequently, the final group sizes differ between conditions. Cognitive performance was assessed using a pre-post-follow-up approach. Pre-testing assessed verbal fluency and attentional performance, followed by a 30-minute intervention. Post-tests encompassed the same measures plus a verbal learning task with immediate recall and interference measures. This verbal learning task was administered only after the intervention to specifically capture acute intervention effects on encoding and interference processes. Delayed recall from the verbal learning task was tested after 20 min and again after 24 h (Fig. 1).

### Participants

Ninety-eight healthy individuals aged between 18 and 37 (mean age ± standard deviation (SD): 23.1 ± 2.8, 54 females) who had obtained at least a German Abitur (equivalent to the high school diploma) were included in the study. Participants were recruited in the environment of the Department Health and Sport Sciences at the Technical University of Munich between 2022 and 2024. Exclusion criteria comprised a history of drug abuse, mental illness, or the use of medications affecting cognitive function. Furthermore, individuals with orthopedic and cardiac conditions that contraindicate participation in the running program or coordinative training were also excluded. The study was approved by the ethics committee of the Technical University of Munich, Germany (case number: 410/21S) and conducted in compliance with the Declaration of Helsinki. All participants provided written informed consent to participate in the study.

### Questionnaire

The German version of the standardized International Physical Activity Questionnaire (IPAQ) (IPAQ Research Committee, 2002) was used to assess self-reported physical activity during a typical week. Participants rated their physical activity on 27 items, including days and time spent on physical activity for transport, work, household tasks, leisure, and sedentary behavior.

We assessed demographic and anthropometric information, including age, highest degree of education, mass, and height, on a self-report basis.

**Table 1:** Participants characteristics across groups

Group:	CG (29)	COOR (37)	HIIT (32)	
Age (years)	23.4 ± 2.9	22.8 ± 2.3	23.1 ± 3.2	^*#*^*p* = .881
Sex (F/M)	17/12	20/17	17/15	^*#*^*p* = .899
Intervention HR average (Hz)	82.4 ± 18.8*	110.0 ± 18.8*	169.0 ± 9.7*	*p* < .001
Intervention HR maximum (Hz)	99.7 ± 20.7*	151.0 ± 24.4*	193.0 ± 9.1*	*p* < .001
IPAQ (MET-min/week)	6961 ± 5718	5474 ± 3564	4616 ± 3503	^*#*^*p* = .190

*F* female, *HR* heart rate, *Hz* Hertz, *IPAQ* The International Physical Activity Questionnaire, *M* male, *MET* metabolic equivalent of task, *min* minutes

^**#**^ = non-parametric test; * = significant difference between the marked group and the other two groups

### Cognitive assessment

#### Verbal fluency task

*Verbal Fluency* is a task that includes a variety of cognitive functions. The participants were initially instructed to name as many words as possible within two minutes, alternating between naming sports and fruits (switching task - VFsw). Subsequently, they were asked to name as many animal names as possible within two minutes (semantic task - VFsem) (Aschenbrenner et al. 2001). The total number of words produced was assessed. Instances where a superordinate category (e.g., bird) was named, followed by its subcategories (e.g., woodpecker), as well as repetitions, were classified as errors.

This task requires vocabulary knowledge, access to semantic information, and retrieval of the lexical representation. Therefore, cognitive functions, such as selective and sustained attention and inhibition, shifting, and self-monitoring, are needed (Patterson 2011). According to the WEAVER + + model proposed by Roelofs and Piai (2011), interference effects in switching tasks can be attributed to the activation of lexical networks associated with specific categories. When words from one category are activated, they must be temporarily stored and inhibited to prevent premature retrieval. This inhibition remains in place until a word from a different, activated category is successfully retrieved and named, leading to increased cognitive load and potential delays in word production due to competition between activated lexical items. The primary outcome measure was the total number of correctly produced words in each of the two tasks.

#### Attention and concentration

To evaluate participants’ attention and concentration abilities, the d2 Test of Attention was administered (Brickenkamp and Zilmer 1998). In this test, participants were presented with a sequence of letters, containing the target letter “d” with one or two dashes above or below it, interspersed with visually similar distractors (“p”). The participants had to scan each row systematically within 20 s and mark only the correctly identified target letters (“d” with two dashes) within the set time limit. After 20 s they had to immediately start with the next row resulting in a total task time of 4:40 min. The d2 test evaluates selective attention, processing speed, and the ability to sustain concentration while filtering out irrelevant stimuli. In addition, speed and accuracy can be measured. As the primary outcome measure, we used the total number of correctly marked symbols.

#### Verbal learning

An adapted version of the California Verbal Learning Test (CVLT) was conducted to evaluate verbal learning, retention, as well as proactive and retroactive interference (Niemann et al. 2009). Four additional items - one per category - were added to the original word list, leading to 20 words in total, trying to reduce ceiling effects in a highly educated young cohort. Performance in the CVLT was measured by counting the number of correctly recalled items for each trial. During the learning phase, participants listened to an audio recording of list A five times, each time followed by an immediate free recall. As an interference task, they then heard list B once, followed by an immediate recall of that list, thereby assessing proactive and retroactive interference. Subsequently, the participants were asked to recall list A again, investigating retroactive interference. To ensure consistent naming speed across subjects, the same audio file was used for all participants, presenting one word per second. After a 20-minute delay, delayed free recall of list A was tested. In addition, a 24-hour delayed recall was administered via telephone call or video conference to test long-term memory retention. Participants were not informed beforehand about the specific content of this follow-up call.

### Intervention protocols

The protocol comprised three groups: two intervention groups and a resting control group. The two intervention groups took part in different types of interventions - high-intensity interval training and coordinative training, while the control group took part in a duration-matched sedentary, low-cognitive activity. For additional details, see supplementary materials. Heart rate was continuously monitored and recorded using a Polar^®^ H10 chest strap during the entire intervention, including warm-up and cool-down phases, for all three groups.

#### High intensity interval training

The 30-minute high-intensity interval training session was performed on a running track. It included a self-paced warm-up and cool-down period of 5 min each, followed by eight 60-second high-intensity intervals with 90 s of active recovery, consisting of walking or slow running. Participants were instructed to run at a steady, maximally sustainable pace during each 60-second interval, ensuring they could maintain this pace across all eight repetitions. A 1-minute interval of high intensity was selected to accommodate participants with varying running experience, enabling them to maintain pace, reach exhaustion, and exert maximal effort. The protocol was designed in accordance with a review from Ai and colleagues (2021), in which the majority of positive effects on cognition were observed following a 20-minute high-intensity cardiovascular exercise bout, which incorporated active rest periods of longer duration than the interval.

#### Coordinative training

The 30-minute coordinative training intervention aimed to target coordination, cognitive flexibility, and motor control through various dynamic tasks. Participants engaged in cognitively enhanced activities involving tossing, catching, balancing, and jumping, responding to both verbal and visual cues. The tasks progressed in complexity, incorporating hand and foot movements, cross-body motions, and multi-tasking challenges. The activities were designed to focus on coordination skills to ensure that the intervention remained low in cardiovascular intensity.

#### Control group

Participants in the control group completed a 30-minute sedentary task involving low- to medium-difficulty Sudoku puzzles, selected as an active but verbally inert control condition that engaged visuospatial-numerical processing while placing minimal demands on the verbal and lexical systems targeted by the present outcome measures (Williams et al. 2026). In prior exercise-cognition research, Sudoku completion did not differ from other sedentary control conditions in memory performance, supporting its suitability as a control task (Blough and Loprinzi 2019). All subjects were familiar with the rules of Sudoku puzzles.

### Statistical analysis

Analyses were carried out with R statistical software (v4.5.1; R Core Team (2025) using several packages (tidyverse (Wickham et al. 2014, 2019)], rstatix (Kassambara 2023b), ggpubr (Kassambara 2023a), data.table (Barrett et al. 2024), Hmisc (Harrell Jr 2025), emmeans (Lenth and Piaskowski 2025), lmerTest (Kuznetsova et al. 2017). All statistical analyses were two-tailed, with the statistical level set at *p* < .05. Unless specified otherwise, data are reported as means ± standard deviations. To aid in interpreting the effect sizes, partial eta-squared (η_p_²) or Cohen’s *d* is indicated. Histograms and normal quantile plots were used to inspect variable distributions for the three groups, the Shapiro-Wilk test was utilized to confirm a normal distribution and the Levene’s test to assess homogeneity of variances for between group comparisons where applicable. Greenhouse–Geisser corrections were applied when assumptions of sphericity were violated. Group differences for absolute values of the demographic variables and baseline performances in the cognitive tests were investigated with one-way analysis of variance (ANOVA), the Kruskal–Wallis test, or the Chi-Square test, depending on the distribution of the data.

For the three outcomes where we assessed performance pre- and post-intervention (namely d2, VFsem, and VFsw), we used 3 × 2 mixed ANOVAs with group (CG, COOR, HIIT; between-subjects) and time (pre, post; within-subjects), using Type-III sums of squares.

Significant interactions were followed by simple effects: within-group pre–post comparisons (paired *t*-tests) and between-group comparisons at post (independent *t*-tests). To directly test whether changes differed between groups, we obtained estimated marginal means (EMMs) from the mixed model, computed change contrasts (post-pre within each group), and compared these changes between groups. When the group × time interaction was not significant, but the main effect of group was, we compared EMMs for groups (averaged over time). Holm adjustment was applied for multiple testing.

Additionally, ANCOVAs with baseline performance entered as a covariate were conducted as a sensitivity analysis for d2, VFsem, and VFsw (see Supplementary Materials S2).

Since the CVLT consists of different domains, such as learning, interference (proactive and retroactive), and retention, we used a series of tests to analyze these components separately. To analyze encoding in the CVLT performance across the five learning trials of list A (A1–A5), we used a 5 (Trial: A1–A5, within-subjects) × 3 (Group, between-subjects) mixed ANOVA. This tested for overall learning effects, group differences, and potential Trial × Group interactions. As a secondary index, the learning slope (per-participant regression slope across A1–A5) was compared across groups using a one-way ANOVA (Baldo et al. 2002). Proactive interference (PI) was assessed in two ways: (i) the raw recall score on List B, compared across groups, and (ii) a proportional PI index defined as (List B−A1)/A1, thereby normalizing List B performance to each participant’s initial encoding (Baldo et al. 2002). Retroactive interference (RI) was likewise evaluated by (i) the raw short-delay recall of List A following List B (Trial A6), and (ii) a proportional RI index defined as (A6 − A5)/A5, reflecting the relative change from the end of learning (Trial A5) to the immediate post-interference recall. Both raw and ratio indices were compared across groups using one-way ANOVAs.

Delayed free recall of List A was assessed at 20 min and 24 h post learning. Raw recall scores were analyzed using one-way ANOVAs for each of the two time points. To control for individual differences in learning, retention ratios were computed as the proportion of delayed recall relative to recall at Trial A5 (i.e., CVLT 20 m/A5 and CVLT 24 h/A5) (similar to (Keith et al. 2023).

## Results

Overall, 98 participants were recruited and allocated to three groups, namely HIIT (*n* = 32), COOR (*n* = 37), and CG (*n* = 29). We did not observe any difference between groups regarding their age, biological sex, or physical activity level according to the IPAQ (Table 1). As expected, the intervention affected the heart rate of participants in the three groups differently. This is evident in both the mean and maximum heart rates during the intervention period, with the HIIT group having the highest values, the CG group the lowest, and the COOR group falling in between. We did not observe statistically significant differences between groups in the baselines of the d2, the two verbal fluency variants, or the first round of the CVLT (Table 2).

**Table 2:** Test performance of the three intervention groups in the pre- and post-assessment and ANOVA effects

Variable	CG (*n* = 29)		COOR (*n* = 37)		HIIT (*n* = 32)		ANOVA effects		
	Pre-test	Post-test	Pre-test	Post-test	Pre-test	Post-test	Interaction	Group	Time
d2 (n)	199.9 ± 40.4	241.0 ± 38.1	194.3 ± 33.2	237.3 ± 35.9	212.6 ± 34.1	261.8 ± 31.5	*p* = .187	*p* = .031*,	*p* < .001*,
								η_p_² = 0.070	η_p_² = 0.859
VFsem (n)	39.3 ± 9.3	43.4 ± 10.0	36.8 ± 8.8	42.5 ± 9.9	38.7 ± 7.8	47.0 ± 8.6	*p* = .029*,	*p* = .297	*p* < .001*,
							η_p_² = 0.072		η_p_² = 0.495
VFsw (n)	25.9 ± 5.0	29.0 ± 5.2	23.8 ± 4.8	28.2 ± 4.9	25.8 ± 4.4	29.4 ± 4.2	*p* = .221	*p* = .267	*p* < .001*,
									η_p_² = 0.580

*CG* control group, *COOR* coordination group, *HIIT* high-intensity interval training group, *d2* d2 test of attention, *VFsem* verbal fluency semantic, *VFsw* verbal fluency switching

*p < .05; Values depicted as means ± SD

**Fig. 2:**
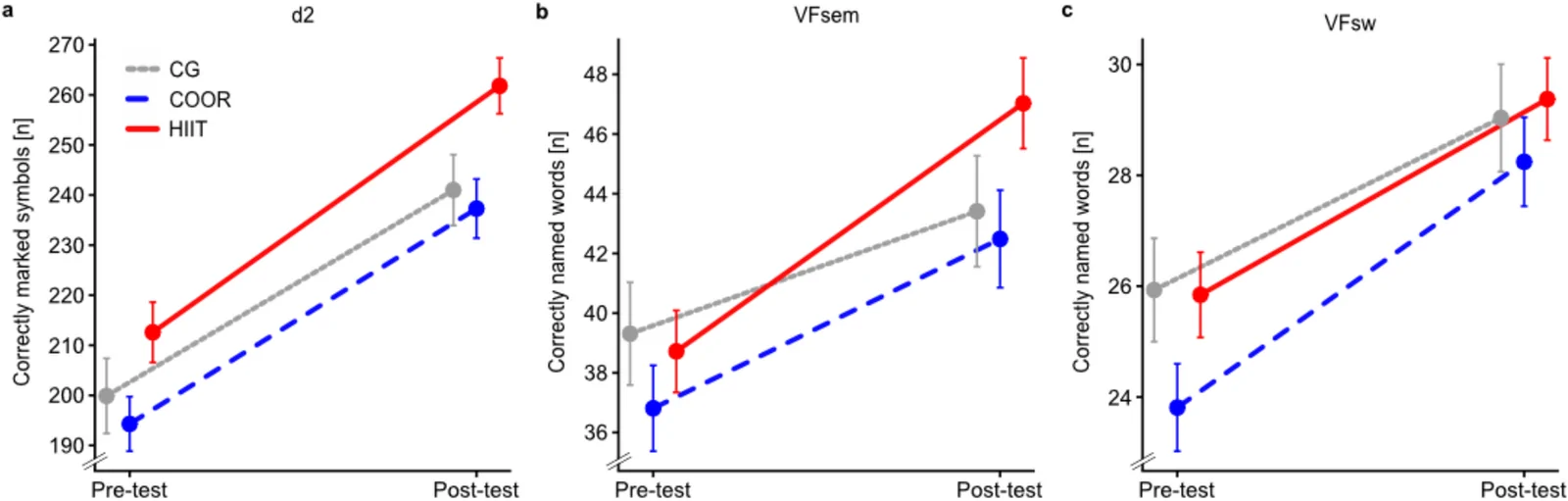
Pre–post changes in cognitive performance across groups. Mean pre- and post-test scores (± SE) for **a** sustained attention (d2), **b** semantic verbal fluency (VFsem), and **c** switching verbal fluency (VFsw) in the control group (CG, grey dotted line), the coordinative training group (COOR, blue dashed line), and the high-intensity interval training group (HIIT, red solid line). While across groups participants improved from pre- to post-test in all three tasks, only for VFsem the interaction of group × time was significant, with post-hoc tests indicating that participants performing the HIIT improved significantly more than the CG

To investigate the influence of the interventions on attention (d2, Fig. 2a; Table 2), we utilized a 3 (group: CG, COOR, HIIT) × 2 (time: pre, post) mixed ANOVA. There was a significant main effect of time (F_(1, 95)_ = 579.08, *p* < .001, η_p_² = 0.859), indicating substantial improvement from pre- to post-test across groups. There was also a significant main effect of group (F_(2, 95)_ = 3.59, *p* = .031, η_p_² = 0.070) while the group × time interaction was not significant (F_(2, 95)_ = 1.71, *p* = .187, η_p_² = 0.035). Holm-adjusted pairwise EMM comparisons revealed that HIIT vs. COOR differed significantly (Δ = 21.41, SE = 8.27, *t*_(95)_ = 2.59, *p* = .034, *d* = 0.44), with the HIIT group performing better overall. The performance of the CG was lying in between and was not significantly different from either COOR or HIIT (*p’s* ≥ .119).

The effect of the interventions on verbal fluency was assessed by testing for differences between groups in the performance of the VFsem and VFsw separately (VFsem and VFsw; Fig. 2b&c and Table 2) using a 3 (group: CG, COOR, HIIT) × 2 (time: pre, post) mixed ANOVA for each of the two test variants. Regarding VFsem, there was a significant main effect of time (F_(1, 95)_ = 93.18, *p* < .001, η_p_² = 0.495) and no main effect of group (F_(2, 95)_ = 1.23, *p* = .297, η_p_² = 0.025). Critically, the group × time interaction was significant (F_(2, 95)_ = 3.68, *p* = .029, η_p_² = 0.072). Using Holm-adjusted within-group comparisons, we observed clear pre–post improvements in all three groups (*p’s* ≤ .002). At post-test, between-group differences did not reach significance after Holm adjustment (CG vs. COOR: *p* = .696; CG vs. HIIT: *p* = .284; COOR vs. HIIT: *p* = .154). Model-based change estimates (EMMs; post − pre) were CG = + 4.10 (SE 1.14), COOR = + 5.68 (SE 1.01), and HIIT = + 8.31 (SE 1.09), all *p* < .001. Pairwise differences of these changes indicated that HIIT improved significantly more than CG (ΔHIIT–CG = 4.21, SE 1.58, t_(95)_ = 2.67, *p* = .027, *d* = 0.70), whereas COOR did not differ significantly from either CG or HIIT (*p’s* ≥ .158).

For VFsw the 3 (group: CG, COOR, HIIT) × 2 (time: pre, post) mixed ANOVA showed a main effect of time (F_(1, 95)_ = 131.25, *p* < .001, η_p_² = 0.580) indicating improvements from pre to post across groups, with no main effect of group (F_(2, 95)_ = 1.34, *p* = .267, η_p_² = 0.027) and no significant group × time interaction (F_(2, 95)_ = 1.54, *p* = .221, η_p_² = 0.031).

Sensitivity analyses using ANCOVA with baseline performance as a covariate yielded consistent results across all three outcomes (see Supplementary Materials S2).

**Fig. 3:**
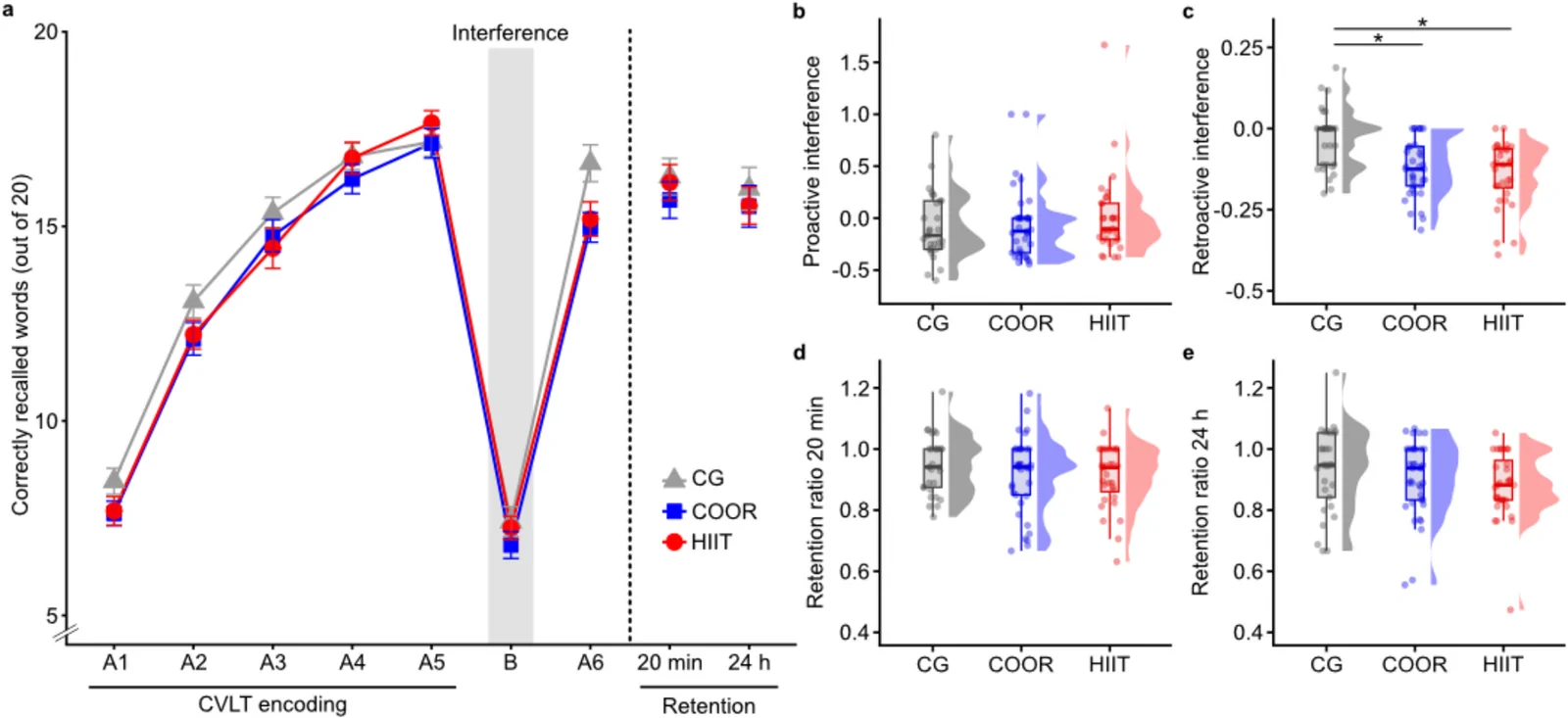
California Verbal Learning Test (CVLT) performance across groups. **a** number of correctly recalled words across trials, with higher values indicating better performance. **b** Proactive interference ((List B − A1)/A1), and **c** Retroactive interference index ((A6 − A5)/A5), where lower values reflect greater interference and more positive values indicate reduced susceptibility to interference. **d** Retention ratio 20 min (20 min/A5) and **e** retention ratio 24 h (24 h/A5), where values below 1.0 indicate forgetting, and values above 1.0 indicate higher delayed relative to immediate recall. The only significant difference between groups was observed in the retroactive interference ((A6 − A5)/A5), with post hoc comparisons indicating that the CG group significantly outperformed both the HIIT and COOR groups, suggesting that both interventions negatively impact retroactive interference in the CVLT. *Indicate that there is a significant difference between groups (*p* < .05)

Learning performance in the CVLT (CVLT trials 1–5, Fig. 3) was assessed using a 5 (trial) × 3 (group) mixed ANOVA. There was a significant effect of trial (F_(3.36, 319.46)_ = 557.56, *p* < .001, η_p_² = 0.854), indicating learning of the word list across repetitions, with no main effect of group (F_(2, 95)_ = 1.01, *p* = .367, η_p_² = 0.021) and no significant group × trial interaction (F_(6.73, 319.46)_ = 1.29, *p* = .258, η_p_² = 0.026). However, the secondary analysis of the learning slope did differ significantly between groups (F_(2, 95)_ = 3.22, *p* = .044, η_p_² = 0.063). Holm-adjusted post-hoc tests revealed that HIIT had a higher slope compared to CG (t_(59)_ = 2.38, *p* = .039, *d* = 0.61), whereas CG vs. COOR (t_(64)_ = -1.67, *p* = .246, *d* = 0.41) and COOR vs. HIIT (t_(67)_ = -1.08, *p* = .280, *d* = 0.26) were not significantly different. This indicates that the intervention influenced the rate of acquisition, with HIIT showing a steeper learning slope than CG, while COOR did not differ significantly from either group.

To investigate the effects of the interventions on proactive and retroactive interference in the CVLT, we compared both the raw recall scores in list B (i.e., proactive interference) and list A6 (i.e., retroactive interference), as well as the proportional indices (List B−A1)/A1 for proactive and (A6 − A5)/A5 for retroactive interference between groups using one-way ANOVAs. Regarding proactive interference, neither the raw List B recall scores nor the proportional proactive interference index differed significantly between groups (List B: F_(2, 95)_ = 0.86, *p* = .428, η_p_² = 0.018; index: F_(2, 95)_ = 0.60, *p* = .552, η_p_² = 0.012). In contrast, we observed significant group differences for both the raw and proportional measures of retroactive interference ((A6 − A5)/A5). For the raw short-delay recall (Trial 6), the ANOVA showed a main effect of group, F_(2, 95)_ = 4.15, *p* = .019, η_p_² = 0.080. Holm-adjusted post-hoc tests indicated that both COOR (t_(64)_ = 2.74, *p* = .024, *d* = 0.68) and HIIT (t_(59)_ = 2.22, *p* = .049, *d* = 0.57) recalled fewer words than the CG, whereas COOR and HIIT did not differ significantly (t_(67)_ = -0.37, *p* = .717, *d* = 0.09). The proportional retroactive interference index ((A6 − A5)/A5) confirmed this pattern, F_(2, 95)_ = 11.64, *p* < .001, η_p_² = 0.197. Post-hoc comparisons indicated that both intervention groups (i.e., COOR and HIIT) exhibited a larger proportional decrease from Trial 5 to Trial 6 than the control group (COOR vs. CG: t_(64)_ = 4.03, *p* < .001, *d* = 0.99; HIIT vs. CG: t_(59)_ = 4.33, *p* < .001, *d* = 1.11), with no significant difference between COOR and HIIT (t_(67)_ = 0.76, *p* = .452, *d* = 0.18).

For the effects of the interventions on 20-minute and 24-hour consolidation in the CVLT, we compared raw recall scores, as well as the retention ratios for both time points, using one-way ANOVAs. For neither the 20-minute nor the 24-hour retention we observed statistically significant differences between groups regarding the raw recall scores (20-minutes: F_(2, 95)_ = 0.46, *p* = .634, η_p_² = 0.010; 24-hours: F_(2, 95)_ = 0.22, *p* = .799, η_p_² = 0.005) or the retention-ratios (20-minutes: F_(2, 95)_ = 0.95, *p* = .389, η_p_² = 0.020; 24-hours: F_(2, 95)_ = 1.33, *p* = .271, η_p_² = 0.027). This indicates that the interventions did not influence the consolidation of the learned word list compared to a sedentary control group.

## Discussion

In recent years, the effects of acute exercise or activity interventions on executive functions and memory processes have received increasing attention (Basso and Suzuki 2017; Cai et al. 2025; Ceylan et al. 2024; Qazi et al. 2024).Yet, there is only sparse existing evidence suggesting beneficial effects on verbal functions, such as language acquisition (Liu et al. 2017; Winter et al. 2007) and language processing (Alatorre-Cruz et al. 2020; Scudder et al. 2014), predominantly following cardiovascular exercise modalities.

However, the overall findings remain mixed, suggesting that the cognitive effects of acute exercise or activity interventions may depend on moderators such as task characteristics, participant features, and modality (Ai et al. 2021; Basso and Suzuki 2017; Cantelon and Giles 2021; Y. K. Chang et al. 2012; Y.-K. Chang et al. 2025; Gothe and McAuley 2015; Li et al. 2017; Ludyga et al. 2020).

The present study investigated the effects of two acute intervention modalities - high-intensity interval training and coordinatively demanding motor activities compared with a sedentary control condition on verbal fluency, verbal learning, and attention in healthy young adults. While practice-related improvements were observed across groups, intervention-specific effects were selective. Acute HIIT enhanced semantic verbal fluency, whereas no group differences emerged for switching fluency or sustained attention. Additionally, in the verbal learning task, we observed a steeper verbal-learning slope for the HIIT compared to the control group, as our secondary learning measure. In both intervention groups, we noted increased retroactive, but not proactive interference, and neither 20 min nor 24 h retention differed between groups.

The improvement in semantic verbal fluency indicates exercise-induced enhancement of lexical retrieval processes. These results are consistent with previous studies, which show that semantic and phonemic verbal fluency improved during and after vigorous cardiovascular exercise (Khanna et al. 2025), and align with the view that high-intensity cardiovascular exercise enhances cerebral blood flow to prefrontal regions and increases catecholamine release, which in turn supports more efficient lexical retrieval as a potential mechanism (Basso and Suzuki 2017). In contrast, we did not observe group differences in the category switching variant of the verbal fluency task (VFsw). Previous research has reported small to moderate effects of acute cardiovascular exercise on global cognitive functioning, particularly regarding cognitive flexibility and inhibitory control (Cantelon and Giles 2021). VFsw draws on a broad set of executive functions, including task switching, and inhibitory control. Successful switching requires the activation of one semantic category, followed by the suppression of the activated semantic network, to access lexical items from the alternate category. The absence of effects in VFsw therefore suggests that, although acute high-intensity cardiovascular exercise facilitated lexical retrieval, it did not sufficiently elevate the executive resources that switching demands.

Regarding the verbal learning data, the primary analysis revealed no significant group x trial interaction. In a secondary analysis, however, we observed a steeper learning slope in the HIIT group compared to the control group. This may potentially reflect a transient facilitation of the encoding processes following high-intensity cardiovascular exercise. This secondary finding should be interpreted with caution, given its exploratory nature, but may indicate that HIIT accelerated the rate of new information uptake, possibly reflecting the transient neurochemical environment associated with acute cardiovascular exercise, including exercise-induced elevations in BDNF and catecholamine levels as reported in prior work (Basso and Suzuki 2017; Szuhany et al. 2015). This is partially consistent with prior evidence linking acute cardiovascular exercise to improved verbal learning (Doku et al. 2025; Patrick et al. 2025), though direct comparisons are complicated because of methodological differences between studies.

An especially noteworthy observation is the increased susceptibility to retroactive interference in both intervention groups compared to the control group. This may suggest that the same neurochemical environment that potentially facilitates encoding could simultaneously increase receptivity to subsequently presented information, thereby leaving recently encoded memory traces more vulnerable to disruption, e.g., through an interference task. Several mechanisms may potentially account for this observation. First, post-intervention fatigue may have transiently reduced the prefrontal inhibitory resources available to suppress the interfering list during recall (Tsukamoto et al. 2022) though this would also predict impairment in proactive interference, which was not observed. Second, intervention-induced increases in arousal and catecholamine release may have intensified competition between the two word lists in memory, making the recently formed List A traces more vulnerable to disruption by the incoming List B material, as is proposed by the GANE model, which posits that norepinephrine, under arousal, amplifies stronger memory representations at the expense of weaker ones (Mather et al. 2016). Third, a timing-related explanation is also plausible, as the entire CVLT, including both List A and the interference List B, was administered within the same post-intervention time window. The interference list may have fallen within a period of heightened synaptic lability, intensifying competition between the two memory traces. Importantly, proactive interference was unaffected across groups, suggesting that the effect is specific to the retroactive direction and arguing against a general impairment of memory control processes.

With respect to the delayed recall in the CVLT, no significant group differences were observed at the 20-minute and 24-hour retention tests. These findings diverge from those reported by Loprinzi (Loprinzi 2018), who observed enhanced 24-hour recall after high-intensity exercise, and others who reported consistently emerging positive effects on memory performance following acute interventions across different exercise and activity modalities (Li et al. 2017), which are attributed to exercise-enhanced long-term potentiation (Loprinzi et al. 2019). These differences may be explained by the inclusion of an interference list, which could have counteracted potential intervention-induced consolidation benefits by heightening susceptibility to retroactive interference observed in both intervention groups. Additionally, given the high performance of the sample, it could be hypothesized that ceiling effects in delayed recall performance may have limited the sensitivity to detect group differences. Furthermore, the transient intervention-induced facilitation of the consolidation process may have already subsided by the time the consolidation process started, given the time that passed between the acute intervention and the verbal learning task.

Together, the verbal fluency and verbal learning findings indicate that acute high-intensity cardiovascular exercise might improve lexical retrieval, particularly under high-intensity conditions, and conceivably suggest a steeper learning slope during encoding as a secondary observation, while simultaneously increasing susceptibility to interference. To examine domain specificity beyond verbal functions, we additionally assessed sustained attention as a non-verbal comparative measure. We observed no significant interaction between group and time, which contrasts with previous research reporting attentional benefits across various exercise and activity modalities. For instance, adolescents demonstrated improved performance on the d2 test following interventions targeting strength, endurance, and coordination compared to a control condition (Altermann and Gröpel 2023). Similarly, earlier work supported these findings, indicating enhanced attentional performance after moderate-intensity exercise compared to imagined activity (Wieland et al. 2022). In the present study, however, we did observe that the HIIT group consistently had a significantly higher performance level compared to the COOR group, with the CG lying in between. Thus, in addition to practice effects, ceiling effects may have emerged within our highly educated and physically fit sample and may have attenuated potential intervention-related gains, especially in the HIIT group. It is worth noting in this context that the intervention-specific effects observed in the present study for the attention and verbal fluency tasks were modest in magnitude compared to the overall improvements from pre- to posttest that we observed in all three groups. This suggests that retest and practice effects constitute a substantial source of variance that should be considered when interpreting the intervention-specific findings.

The collective pattern of results of studies in humans and rodents suggests that acute exercise and activity interventions yield selective rather than uniform cognitive effects. These effects are thought to arise from neurophysiological and neurochemical pathways. Targeting cardiovascular exercise, neurophysiologically, EEG-, fNIRS-, fMRI-, and TMS-based evidence indicate increased cerebral blood flow, particularly in frontal regions, and exercise-induced modulation of neural oscillations. Enhanced hippocampal theta activity and increases in delta, theta, and alpha power across the cortex have been interpreted as suggesting a state of relaxed alertness, improved attentional regulation, and are closely linked to learning and cognitive control in prior work (Basso and Suzuki 2017).

Neurochemically, acute high-intensity cardiovascular exercise has been associated with elevations in lactate levels, which are believed to act as signals for an increase of key neuromodulators and neurotrophins, most notably BDNF, which supports synaptic plasticity and memory formation (Basso and Suzuki 2017; Szuhany et al. 2015). Additional changes reported in the literature include increases in cortisol, IGF-1, VEGF, and several neurotransmitters (e.g., dopamine, norepinephrine, serotonin). While high-intensity cardiovascular exercise can elevate cortisol to levels that impair memory performance (Basso and Suzuki 2017; Cantelon and Giles 2021; Ferrer-Uris et al. 2022), BDNF expression tends to rise with intensity, consistent with dose-response models of exercise-induced cognitive enhancement (Basso and Suzuki 2017). As none of these neurochemical markers were assessed in the present study, we cannot determine their specific contribution to the observed effects. Nevertheless, our findings partially align with both perspectives, suggesting that high-intensity cardiorespiratory exercise may simultaneously be associated with impaired memory performance, possibly linked, in line with prior literature, to elevated cortisol levels, and enhanced lexical retrieval consistent with previously reported intensity-dependent BDNF responses.

In contrast, the mechanisms underlying coordinatively engaging motor activity remain less clearly defined and more hypothesis-driven. The *cognitive stimulation hypothesis* postulates that cognitive-demanding training leads to improved cognitive functions by engaging similar networks. Accordingly, coordinative training should rather lead to cognitive improvements than solely aerobic training due to the use of similar cognitive demands and neural networks (Ludyga et al. 2020; Pesce et al. 2021; Ringenbach et al. 2021). Recent work on physical activity complexity and cognitive-motor entrainment suggests that memory-related benefits of motor activity may depend not only on the cognitive demands of the movement itself, but also on how motor and cognitive processes are temporally coordinated during learning (Schmid et al. 2023; D. G. Schmid 2025).

The non-significant findings for the coordinative intervention condition are open to at least two interpretations: the cognitive demands of the present protocol may not have been challenging enough to elicit a measurable effect, or alternatively, coordinatively engaging motor activity may not produce meaningful modality-specific enhancements in verbal functions and attention beyond the effects of the cardiovascular component. Additionally, we cannot draw definite conclusions about the influence of the utilized coordinative protocol compared to solely cardiovascular intervention of similar intensity. Beyond modality, HIIT and the coordinative intervention conditions differed in physiological intensity and probably also in arousal state, fatigue, and motivational experience. None of those were systematically assessed in this study. Therefore, it remains unclear whether the observed HIIT-specific effects reflect differences in modality per se, intensity-dependent physiological differences, modality-specific psychological factors, or a combination of these.

Although these mechanisms help explain how acute exercise or activity may influence cognition, in our case, verbal functions and attention, their expression depends on several moderating factors, including intervention intensity. A recent meta-review of meta-analyses identified an inverted U-shaped relationship between exercise intensity and cognitive performance (e.g., attention, executive functions, memory, information processing), indicating that moderate-to-high intensities yield the greatest benefits, whereas low-intensity exercise produces negligible effects (Y.-K. Chang et al. 2025). Accordingly, maybe neither the HIIT nor the COOR (low-intensity, despite potentially other pathways) protocol targeted optimal intensities, since recent studies favoring moderate intensities (Cantelon and Giles 2021; Herbert 2025), which may have contributed to the discrepant results. In the context of intervention modality, contrary to our findings, cognitive benefits were found to be largely independent of intervention modality, with the most robust improvements emerging after cardiovascular and also combined interventions that integrate stress-reduction practices such as yoga or tai chi. The effects of combined interventions might rely on the regulation of the sympathetic nervous system and the hypothalamic-pituitary adrenal (HPA) axis to regulate stress (Y.-K. Chang et al. 2025; Gothe and McAuley 2015).

Considering these existing moderators, the question arises whether the observed effects stem from a general enhancement of alertness or from task-specific improvements. Evidence indicates global cognitive enhancement following acute interventions, independent of modality, with small to moderate effects on executive functions, attention, and smaller effects on memory (Y.-K. Chang et al. 2025), which might rather indicate higher alertness than task-specific effects. In contrast, our findings suggest task-specific effects rather than a broad, unspecific boost.

Taken together, coordinative motor activity, beyond being a physically engaging but low-intensity motor task, does not induce modality-specific enhancements in verbal functions, verbal learning, or sustained attention performance in our cohort. More generally, our findings contribute to the relatively little explored area of the acute effects of exercise or activity on verbal functions in healthy young adults, despite extensive research on exercise, activity, and cognition.

The present findings offer novel insights into how specific intervention modalities, including high-intensity cardiovascular exercise and coordinatively demanding motor activity, influence lexical-semantic processing, affect the learning slope, and contribute to memory interference, while having no effect on attention after acute interventions. This suggests an intensity-dependent pattern, characterized by temporarily enhanced lexical retrieval and increased receptivity to new information, accompanied by a transient vulnerability to interference. However, several questions remain unanswered. For instance, the duration of the observed effects following exercise or activity interventions, the reproducibility of this pattern, and the potential influence on interference processes warrant further investigation.

Overall, the results suggest that acute physical activity interventions partially enhance verbal functions, with effects varying based on intervention modality. Notably, the observed improvements in lexical retrieval and encoding efficiency were accompanied by increased susceptibility to retroactive interference, pointing to a nuanced interplay between intervention-induced cognitive facilitation and vulnerability to disruption.

### Limitations

We have to acknowledge that there are some limitations inherent to this study. First, we recruited a highly educated cohort in the environment of the Department Health and Sport Sciences. Therefore, it can be assumed that participants are accustomed to physical activity and exercise, suggesting that long-term exercise adaptations may moderate or mask acute effects, making them less pronounced. In contrast, studies with trained individuals revealed positive effects of acute cardiovascular exercise on cognition, which may be restricted to the task type rather than, according to our results, a broad, unspecific boost (Browne et al. 2017; Niedermeier et al. 2020). Additionally, due to participants’ high cognitive level, we hypothesize that ceiling effects may have emerged in the d2 test of attention, and potentially also in the CVLT. Another limitation concerns the potential influence of retest or practice effects on the observed outcomes in the attention and verbal fluency tasks. Although the group × time interaction design statistically controls for practice effects shared across groups, future studies might consider approaches to reduce the contribution of repeated-testing variance. For instance, incorporating familiarization sessions, when feasible given the characteristics of the measures employed, could help to bring participants closer to the performance asymptote before introducing intervention conditions. Furthermore, the cognitive assessment after the 30-minute intervention took approximately one hour. Acute effects could be attenuated with increasing test duration due to the unfavorable ratio of intervention to test (see e.g. Y. K. Chang et al. (2012) or the temporal distance to the intervention. Some studies, however, could not observe differences in delay times in cognitive testing of up to 2 h in effects on prefrontal cortex-dependent cognition (Basso et al. 2015). Additionally, studies revealed improvements in cognitive functions across different exercise or activity modalities (Chen et al. 2023; Gothe and McAuley 2015; Ludyga et al. 2020). Still, we cannot draw definite conclusions about the influence of the utilized coordinative training intervention compared to a solely cardiovascular intervention of similar intensity, and thus untangle the differences in effect between aerobic and cognitively stimulating interventions. As noted in the discussion, the HIIT- and COOR-groups likely differed across multiple dimensions beyond modality, including arousal, fatigue, and motivational experience. This limits the interpretability of between-condition differences and, in turn, conclusions about the specific drivers of the observed effects. Future studies should consider including comparison conditions matched for intervention intensity to better isolate the specific contribution of modality to cognitive outcomes.

## Conclusion

To conclude, our study revealed task-specific improvements in verbal functions following HIIT. The intensity of the intervention plays a crucial role in shaping cognitive outcomes; we observed that HIIT has the potential to enhance lexical retrieval and, as a secondary exploratory finding, is associated with a steeper learning slope in the CVLT. Interestingly, we found that retroactive interference in this task was heightened in both intervention groups. Taken together, these findings point to a transient, intensity-dependent state of heightened receptivity to incoming information, potentially leading to increased vulnerability to interference.

## Supplementary Information

Below is the link to the electronic supplementary material.

Supplementary Material 1

## Data Availability

The datasets generated during and/or analyzed during the current study are available from the corresponding author upon reasonable request.
