# Supplementary material for: Effects of acute cardiovascular exercise and coordinative motor activity on verbal functions and attention
Source: Cogn Process. 2026 Jul 29;27(4):1285–98. doi: 10.1007/s10339-026-01380-2 (PMC13714002; doi:10.1007/s10339-026-01380-2)
Supplement: Supplementary file 1 [file 10339_2026_1380_MOESM1_ESM.pdf]

Effects of acute cardiovascular exercise and coordinative motor activity on verbal functions and attention | Supplementary Materials

S1 Detailed Description of the Intervention Protocols

S1.1 General Intervention Characteristics

All intervention sessions lasted 30 minutes and were conducted in a supervised setting. Participants were randomly assigned to one of three conditions: Control group (CG), High-Intensity Interval Training (HIIT), or Coordination Training (COOR). All interventions were delivered alone or in pairs. Instructions were standardized and provided by trained staff members.

Equipment

Used in all three intervention groups:

- Polar H10 heart rate chest strap
- Polar M430 watch

S1.2 Control Condition

Purpose

Passive control condition with minimal physical and cognitive demands.

Procedure

Participants were instructed to remain seated for the entire 30-minute session. To prevent boredom without inducing substantial cognitive load, participants were provided with easy Sudoku puzzles. The use of the Smartphone was prohibited during the 30-minute intervention. The intervention was conducted indoors in the laboratory.

Equipment

- Printed Sudoku sheets and pens.

S1.3 High-Intensity Interval Training (HIIT)

Purpose

To induce vigorous cardiovascular exertion through a standardized HIIT protocol.

Equipment

- automated external defibrillator (AED) available on site.

Personnel

Two staff members were present throughout each session. At least one staff member held a valid and regularly refreshed Basic Life Support (BLS) certification.

Protocol Structure

Total duration was 30 minutes, consisting of a 5-minute warm-up with light running, a 20-minute interval phase, and a 5-minute cool-down with walking or light jogging. The intervention was carried out on the 400 m tartan track located at the Sports and Health Campus of the Technical University of Munich.

Interval Phase

The interval phase consisted of eight blocks. Each block consisted of 60 seconds of high-intensity running, followed by 90 seconds of active recovery, which included slow running or walking.

Intensity Regulation

Participants were instructed to perform the 60-second intervals at a very intense but sustainable pace, primarily based on perceived exertion. Heart rate was used as an orientation aid, with a target of approximately 93% of the age-predicted maximum heart rate (HRmax), calculated using the Karvonen formula 220 minus age. Participants were informed that this value represents a rough estimate and may vary due to individual differences and measurement error.

Instructions to Participants

Participants were instructed that the chosen pace should be maintainable for the full 60-second interval. Heart rate values were intended as guidance rather than strict thresholds. Participants were encouraged to adjust their pace as needed.

S1.4 Coordination Training

Purpose

Coordinatively demanding motor activity combining balance, bilateral coordination, hand–eye coordination, and cognitive-motor interference, with low cardiovascular load.

Materials

- Beanbags
- Floor rope (laid flat on the ground)
- Balls (e.g., soft balls suitable for dribbling and balancing)
- Juggling scarves
- Books (used as balancing objects)
- Stopwatch or timer for station timing

General Structure

The total duration was 30 minutes. The training consisted of six stations, with approximately five minutes spent at each station. Each exercise was conducted for one or two minutes, respectively, and included different variations with increasing difficulty. Participants rotated sequentially through all stations. The intervention was carried out indoors.

Station 1: Beanbag Throwing and Catching

Participants performed throwing and catching tasks with a partner using beanbags. Initially, hand movements were congruent with verbal instructions, followed by incongruent hand movements. In later stages, participants coordinated their footsteps according to partner cues and responded to visual hand signals while catching the beanbag.

Station 2: Crossed-Hand Catching

Participants threw a beanbag into the air and attempted to catch it using crossed hands, requiring bilateral coordination and spatial awareness.

Station 3: Elephant Exercise

Participants performed a sequence of coordination tasks involving alternating hand positions, with one hand touching the nose and the other the ear, followed by repeated switching. Task complexity was increased by adding clapping movements between switches, including clapping hands together and clapping on the lap.

Station 4: Balancing on a Rope

Participants walked along a rope laid on the floor. Task difficulty was progressively increased by adding ball dribbling and a figure-eight movement performed with a juggling scarf.

Station 5: Balancing with Books and Balls

Participants balanced a book on their head and held an additional book in one hand, each with a ball placed on top of it. While walking, participants were required to maintain balance and stability.

Station 6: Jumping Sequence with Counting

Participants jumped across a rope in a predefined sequence while simultaneously counting aloud. Counting tasks alternated between simple number sequences and mixed number–letter sequences to increase cognitive load.

S1.5 Safety and Ethical Considerations

All procedures were conducted in accordance with the principles of the Declaration of Helsinki. The study was non-invasive and painless, and no short- or long-term adverse effects were expected.

Risk Assessment

The physical demands of the HIIT condition corresponded to vigorous physical activity. According to the American College of Sports Medicine (ACSM), the risk of adverse cardiovascular events during vigorous exercise in individuals without known cardiovascular disease is extremely low. The coordination training involved low cardiovascular strain and was not associated with increased health risks.

Participant Information and Screening

All participants were informed about potential risks prior to participation. Written informed consent was obtained, and participants confirmed the absence of cardiovascular, orthopedic, metabolic, or other contraindications.

S2 Sensitivity analysis: ANCOVA with baseline as covariate

S2.1 Statistical analysis

As a sensitivity analysis, we analyzed the effects of the interventions on attention (d2), semantic verbal fluency (VFsem), and category-switching verbal fluency (VFsw) using analysis of covariance (ANCOVA), with post-intervention scores as the dependent variable, group as the between-subjects factor, and baseline (pre-intervention) scores as the covariate. Prior to each ANCOVA, we verified the assumption of homogeneity of regression slopes by testing the group × baseline interaction. In all three cases, this interaction was non-significant (*p's* ≥ .408), confirming the appropriateness of the standard ANCOVA model. Normality of residuals was confirmed via Shapiro-Wilk tests (*p's* ≥ .094) and homogeneity of variance via Levene's tests (*p's* ≥ .444). Pairwise contrasts between groups were based on baseline-adjusted post-intervention means (estimated marginal means) and were Holm-adjusted for multiple comparisons.

S2.2 Results

Regarding d2, the ANCOVA revealed no significant group effect after controlling for baseline performance ($F_{(2, 94)} = 2.96$, $p = .057$, $\eta_p^2 = .059$). Holm-adjusted pairwise contrasts showed no significant differences between any group pairs (all *p's* ≥ .093), consistent with the non-significant group × time interaction in the primary analysis.

For VFsem, there was a significant group effect after controlling for baseline performance ($F_{(2, 94)} = 3.80$, $p = .026$, $\eta_p^2 = .075$). Holm-adjusted pairwise contrasts indicated that HIIT showed significantly higher adjusted post-intervention scores than

CG ($t_{(94)}$ = 2.65, *p* = .028, *d* = 0.68), while COOR did not differ significantly from either CG ($t_{(94)}$ = 0.80, *p* = .423, *d* = 0.20) or HIIT ($t_{(94)}$ = 1.98, *p* = .102, *d* = 0.48), with COOR lying in between. These findings are fully consistent with the mixed ANOVA results reported in the main manuscript.

Regarding VFsw, the ANCOVA revealed no significant group effect ($F_{(2, 94)}$ = 0.66, *p* = .519, η_p^2 = .014), and no pairwise contrasts reached significance (all *p's* ≥ .765), fully consistent with the primary mixed ANOVA.
